# Genetic inactivation of FAAP100 causes Fanconi anemia due to disruption of the monoubiquitin ligase core complex

**DOI:** 10.1172/JCI187323

**Published:** 2025-04-15

**Authors:** Julia Kuehl, Yutong Xue, Fenghua Yuan, Ramanagouda Ramanagoudr-Bhojappa, Simone Pickel, Reinhard Kalb, Settara C. Chandrasekharappa, Weidong Wang, Yanbin Zhang, Detlev Schindler

**Affiliations:** 1 Department of Human Genetics, Biozentrum, University of Wurzburg, Wurzburg, Germany.; 2 Laboratory of Genetics and Genomics, National Institute on Aging, Baltimore, Maryland, USA.; 3 Department of Biochemistry and Molecular Biology, University of Miami Miller School of Medicine, Miami, Florida, USA.; 4 Genomics Core and Cancer Genomics Unit, Cancer Genetics and Comparative Genomics Branch, National Human Genome Research Institute (NHGRI), NIH, Bethesda, Maryland, USA.

**Keywords:** Cell biology, Genetics, DNA repair, Genetic instability, Monogenic diseases

## Abstract

The Fanconi anemia/breast cancer (FA/BRCA) DNA repair network promotes the removal of DNA interstrand crosslinks (ICLs) to counteract their devastating consequences, including oncogenesis. Network signaling is initiated by the FA core complex, which consists of 7 authentic FA proteins and an FA-associated protein, FAAP100, with incompletely characterized roles and unknown disease associations. Upon activation, the FA core complex functions as a multiprotein E3 ubiquitin ligase centered on its catalytic module, the FANCB-FANCL-FAAP100 (BLP100) subcomplex, for FANCD2 and FANCI monoubiquitylation. Here, we identified a homozygous variant in *FAAP100*, c.1642A>C, predicting p.(T542P), in a fetus with malformations suggestive of FA. The mutation caused sensitivity to ICL-inducing agents in cells from the affected individual and genetically engineered, FAAP100-inactivated human, avian, zebrafish, and mouse cells. All FAAP100-deficient cell types were rescued by ectopic expression of WT FAAP100, but not FAAP100^T542P^. In a confirmatory animal model, customized *Faap100^–/–^* mice exhibited embryonic lethality, microsomia, malformations, and gonadal atrophy resembling mice with established FA subtypes. Mechanistically, FAAP100^T542P^ impaired ligase activity by preventing BLP100 subcomplex formation, resulting in defective FAAP100^T542P^ nuclear translocation and chromatin recruitment. FAAP100 dysfunction that disrupted the FA pathway and impaired genomic maintenance, together with FA-consistent human manifestations, recommends *FAAP100* as a legitimate FA gene, alias *FANCX*.

## Abstract 2

In conclusion, we have identified and characterized a homozygous pathogenic variant in *FAAP100* that leads to FA, justifying the proposal of the alias *FANCY*.

## Graphical abstract

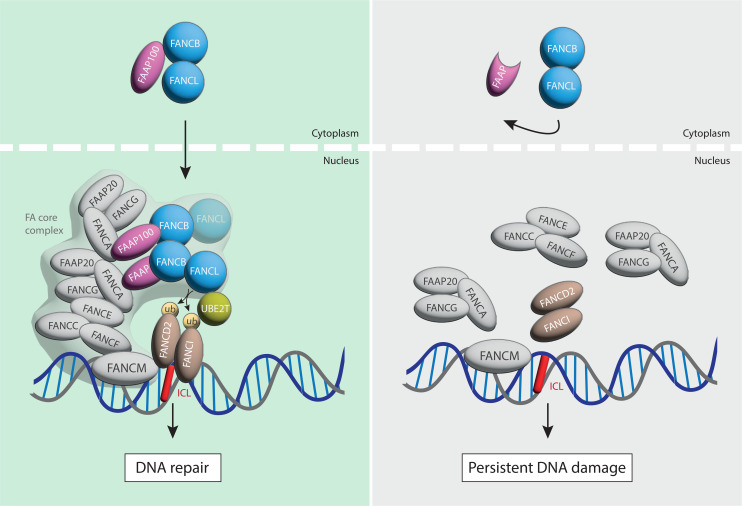

## Introduction

DNA integrity is vital for the faithful replication of genomic information and the regulated initiation of DNA-dependent downstream cellular processes. In higher organisms, maintaining genome intactness is also essential to thwart malignant transformation, prevent premature cellular senescence, and protect stem cells (1, 2, 3). Highly specific biochemical processes, collectively referred to as DNA damage detection, signaling, and repair pathways, serve this purpose. When they fail due to genetic defects, DNA repair disorders occur. One such disease is Fanconi anemia (FA), a rare, mostly recessive disorder (4) with variable features but a recognizable pattern of collectively characteristic clinical phenotypes. Frequent manifestations include developmental abnormalities — most commonly growth retardation, skin pigment displacement, microphthalmia and microcephaly, radial ray defects, typical brain and inner organ malformations, and reduced fertility. Other common features of FA include early-onset bone marrow failure and a predisposition to cancer, particularly acute myeloid leukemia and head and neck (oropharyngeal) squamous cell carcinoma (5). FA results from defects in the FA/breast cancer (FA/BRCA) network, which plays an essential role in the repair of DNA interstrand crosslinks (ICLs), which are highly cytotoxic covalent bonds between the sister strands that impede replication and transcription (6). A large number of genes and proteins are required to sense ICLs, orchestrate strand cleavage at sites of crosslinked DNA, and mediate DNA repair transactions; corresponding single gene defects are reflected in the FA subgroups (7). Common to all subtypes is the cellular sensitivity to endogenously or exogenously induced ICLs, which manifest as spontaneous and induced chromosome breakage, arrest in the G_2_ phase of the cell cycle, and reduced cell survival (8). The FA/BRCA network includes the products of 22 genes identified collectively with the prefix *FANC* and followed by the letters A through W, in alphabetical order (9). Germline inactivation of any one of the *FANC* genes results in FA of the corresponding subtype with classical FA or variant (FA-like) disease, the latter lacking single clinical hallmarks of FA. *FANCM* was assigned the prefix *FANC* before biallelic pathogenic variants in *FANCM* were shown much later to be associated with cancer but not with physical FA phenotypes (10, 11).

Upon recruitment to ICLs, 8 proteins (FANCA, FANCB, FANCC, FANCE, FANCF, FANCG, FANCL, and FAAP100) assemble from partially preformed subunits to form the FA core complex, which represents the initiation step of the FA network. The FA core complex consists of a catalytic module (FANCB-FANCL-FAAP100, hereafter designated BLP100) and 2 other subcomplexes (FANCA-FANCG and FANCC-FANCE-FANCF) with nonredundant and complementary functions in chromatin recruitment and substrate recognition, respectively (12). Assembly of 2 subunits of BLP100 occurs as a FANCB-mediated dimer of trimers at the center of the FA core complex, which stabilizes each member and the entire complex and protects them from proteolytic degradation (13). Cryogenic-sample electron microscopy (cryoEM) revealed a structure of the chicken FA core complex (without FANCA), in which the 2 BLP100 heterotrimers act as a scaffold for the assembly of the remaining subunits, resulting in an extended asymmetric structure (14). The FA core complex with the ubiquitin E3 RING ligase activity containing FANCL and the ubiquitin E2 ligase UBE2T (FANCT) are critical for the targeted and coordinated monoubiquitylation of ataxia telangiectasia and Rad3-related protein (ATR) phosphorylated FANCD2 (D2) and FANCI (I) in a single entity, a key activation event in the FA/BRCA pathway. Monoubiquitylation results in the retention of the ID2 complex on DNA (15). Recent work has confirmed the asymmetric assembly of the human FA core complex with 2 copies of all subunits except the FANCC-FANCE-FANCF subcomplex to leave a space for the E2 ubiquitin ligase UBE2T/FANCT, with a single FANCL-UBE2T cascade monoubiquitylating FANCD2 and FANCI sequentially, converting the ID2 complex conformation into a sliding DNA clamp (16).

In 2003, one of us purified a multiprotein complex called BRAFT, consisting of the Bloom syndrome (BS) complex and the FA core complex (17). In the latter, the 5 then-known FA proteins were identified, namely FANCA, FANCC, FANCE, FANCF, and FANCG. In addition, several previously unknown proteins were discovered and named FA-associated proteins, including FAAP300 (later FAAP250), FAAP100, FAAP90 (later FAAP95), FAAP75, and FAAP43 according to their molecular weights. Subsequently, as patients with FA with pathogenic variants in the corresponding genes were identified, FAAP43 was renamed FANCL (18), FAAP95 was renamed FANCB (19), and FAAP250 was renamed FANCM (20). FAAP75 was renamed BLAP75 and, finally, RMI1, an essential component of the BS complex (21). Later, other FAAPs that are not constitutional components were copurified with the FA core complex, such as FAAP10, FAAP16, FAAP20, and FAAP24. These proteins are not essential for FANCD2 monoubiquitylation or for the structural integrity of the FA core complex. The transiently FANCA-associated protein FAAP20, for example, contains a UBZ domain that preferentially binds the ubiquitin product of RNF8-UBC13 in the vicinity of ICLs to recruit the FA core complex through its interaction with FANCA and coactivate the FA pathway in a replication-independent manner (22). FAAP100 was identified as a critical component of the FA/BRCA DNA damage response network, essential for the stability and function of the complex and was shown to interact with FANCB and FANCL in a stable subcomplex already upon discovery (23). There was no further functional characterization until recently, when FAAP100 was shown to play an essential role in R‑loop resolution and replication fork protection (24). To date, FAAP100 remains the only member of the FA core complex that has not been implicated in human disease. Here, we report that human FAAP100 deficiency causes FA and provide supporting evidence from multiple cellular and animal models.

## Results

### A variant in FAAP100 reveals an association with FA.

**Figure 1:**
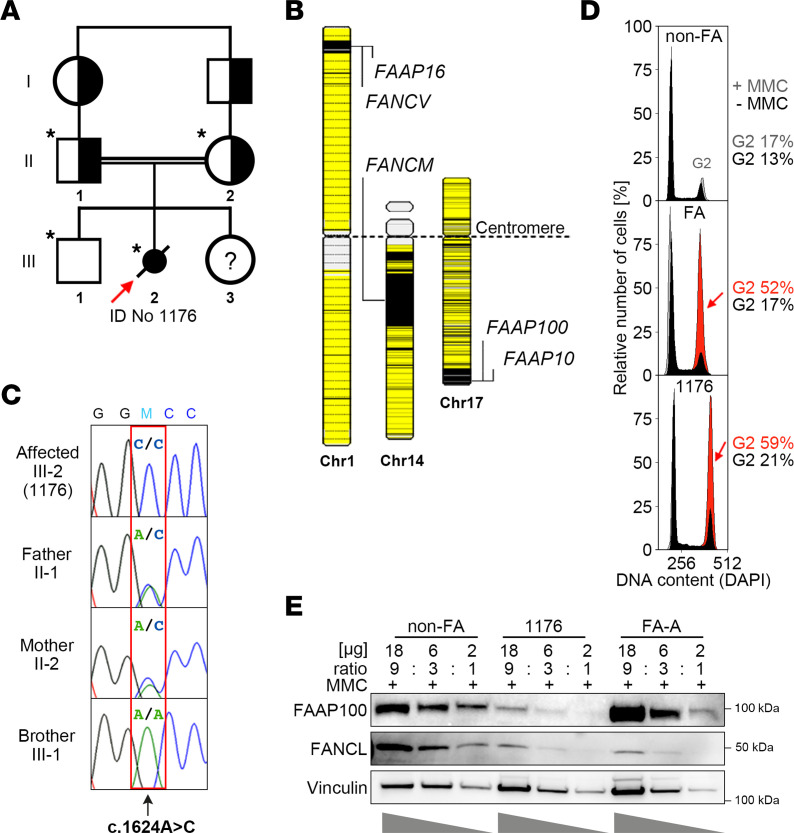
Origin, location, segregation, and implications of the *FAAP100* variant. (**A**) Pedigree of a fetus with FA-suggestive phenotype (III-2, affected individual; solid and slashed circle, marked with a red arrow, ID no. 1176) and family. Squares denote males; circles denote females. Genotyped individuals of the core family are indicated by an asterisk, unassessed genotypes by a question mark, and proven or obligate heterozygotes by half-filled symbols. (**B**) Five FA (prefix *FANC*) and FA-associated (prefix *FAAP*) genes in major autozygous regions (black blocks) on 3 different chromosomes (Chr) in the affected III-2 (1176) are shown. (**C**) Sanger electropherograms identified a homozygous A>C variant sequence, heterozygous sequence, or WT sequence at *FAAP100* position c.1624 (highlighted by a red frame), predicting p.(T542P), in relatives of the core family as indicated. (**D**) Cell-cycle analysis of cultured amniotic fibroblasts by flow cytometry. Exemplary individual measurements. Histograms of cells from a normal control (non-FA; top), a fetus with FA (positive control; middle), and fetus III-2 (1176; bottom). Black histograms are from untreated cultures and superimposed gray or gray/red histograms are from cultures exposed to MMC (10 ng/mL, 48 hours), shown individually in Supplemental Figure 1C. The percentage of cells in G_2_ phase is indicated. Red coloring and arrows denote an increased G_2_ compartment size. Variability and significance of G_2_ phase arrest in repeated measurements are shown in Supplemental Figure 4A. (**E**) Titration experiments show reduced levels of FAAP100 protein in *FAAP100^T542P^*-mutant 1176 cells from affected III-2 cells (approximately 3 steps of 1:3 dilution each lower than in non-FA cells). Reduced levels of FANCL are also suggested in 1176 cells (approximately 2 steps of 1:3 dilution lower than in non-FA cells). Wedges represent dilutions at the ratios indicated above the blots. FA-A, cells from an individual with FA, subtype A. Vinculin was used as a loading control.

We investigated the genetic causes of rare individuals with unclassified FA-like phenotypes. Seven DNAs from cell lines deficient in FANCD2 monoubiquitylation were included. SNP mapping in 1 DNA originating from a fetus (III-2, ID no. 1176), offspring of first cousins, revealed 15 autozygous regions of greater than 0.2 Mb in size (Figure 1A and Supplemental Table 1; supplemental material available online with this article; https://doi.org/10.1172/JCI187323DS1). Seven of them contained a total of 20 genes associated with DNA repair, including 3 regions with the FA and FA-associated genes *FANCM*, *FANCV*, *MHF1*/*FAAP16*, *MHF2*/*FAAP10*, and *FAAP100* (Supplemental Table 1 and Figure 1B). After filtering and prioritization, whole-exome sequencing (WES) results identified a conspicuous change in *FAAP100* (Supplemental Figure 1A). Sanger sequencing confirmed the homozygous sequence variant NM_025161.6(FAAP100):c.1624A>C, rs1598596062, chromosome position 17-81547458T>G (GRCh38) (Supplemental Figure 1B). It segregates in this family, consistent with an autosomal recessive trait (Figure 1C). No other DNA repair–related genes in the major autozygous regions were found to have disease-causing variants, including none in the 4 FA or FA-associated genes *FANCM*, *FANCV*, *MHF1*/*FAAP16*, or *MHF2*/*FAAP10* that were excluded by sequence analysis in the WES. The WES also failed to detect homozygous pathogenic variants in DNA repair genes outside of these regions. The functions of 19 genes with homozygous variants in exons or at canonical splice sites that were predicted to be potentially deleterious were not related to nuclear DNA repair according to current knowledge (Supplemental Table 2). Meanwhile, the abnormalities in the fetus of interest, including growth retardation, radial ray defects, duodenal atresia, ventricular septal defect, and hydrocephalus (Supplemental Figure 2), as well as the cellular hypersensitivity to ICL induction by mitomycin C (MMC), as determined by the disproportionate accumulation of the amniocytes in G_2_ phase on flow cytometric cell-cycle analysis (Figure 1D and Supplemental Figure 1C), were consistent with characteristic manifestations of the FA spectrum. Because of the severe malformations, the pregnancy was discontinued.

### Expression and variant effect prediction in silico analyses suggest impaired stability and function of the FAAP100 variant.

c*.*1624 occurs in all *FAAP100* transcript variants, and c.1624A>C is expressed in *FAAP100* transcripts of the fibroblast line 1176 with the homozygous genomic variant, derived from the affected fetus (no. 1176) at 21+1 weeks of gestation (Supplemental Figure 1, D and E). The expression level of *FAAP100* transcripts in 1176 cells was slightly lower than in a normal control fibroblast line (Supplemental Figure 1E). The presence of the mutation was confirmed in *FAAP100* cDNA from 1176 cells (Supplemental Figure 1E). *FAAP100* c.1624A>C results in the threonine-to-proline substitution p.(T542P) (NP_079437.5:p.(Thr542Pro)). T542P-mutant FAAP100 (FAAP100^T542P^) protein is present in 1176 cells at reduced levels compared with WT FAAP100 (FAAP100^WT^) in control and FA-A cells, which was determined by titration to be approximately 1/27 of normal (Figure 1E, and see also the corresponding blots below). FANCL protein also appears to be present at lower levels in FAAP100^T542P^-mutant 1176 fibroblasts at approximately one-ninth of normal (Figure 1E), whereas, conversely, FAAP100 protein levels are reduced in FANCL-deficient cells (23). Furthermore, lower levels of FAAP100^WT^ are suggested in an FA-B line with the hemizygous pathogenic variant in *FANCB* c.832C>T predicting p.Q278* (25) compared with non-FA or FA-D2 cells (compare ratios on the corresponding blots below), suggesting destabilizing effects resulting from defects in the molecular interaction of FAAP100, FANCB, and FANCL. T542 is located in a region identified as belonging to a shared protein family named FANCAA (beyond amino acid position 448) (http://pfam.xfam.org/; https://www.ebi.ac.uk/interpro/; Supplemental Figure 1D and Supplemental Figure 3A), but not in a known functional motif or domain. T542 is highly conserved in vertebrates, but not in other organisms, and is surrounded in vertebrates by a block of also-conserved amino acids (Supplemental Figure 3B). p.T542P is unknown to most databases of normal or disease-associated human genetic diversity, including the gnomAD platform, version 4.1.0 (https://gnomad.broadinstitute.org/), ClinVar (https://www.ncbi.nlm.nih.gov/clinvar/), and other large-scale reference human genetic variation datasets (see Methods). However, it has received an entry in the NCBI dbSNP database (https://www.ncbi.nlm.nih.gov/snp/), reflecting its single observation in the Korean Genome Project (Korea1K) (26). In silico analyses using, among others (see Methods), Alamut Visual Plus, version 1.12 (https://www.sophiagenetics.com/sophia-ddm-for-genomics/alamut-visual-plus/), and AlphaMissense (https://alphamissense.hegelab.org/) classify the substitution as a variant of unknown significance or likely pathogenic, respectively. Structural modeling shows that the threonine at position 542 is part of a β-strand within an antiparallel β-sheet in a region of the protein with very high structural confidence (Supplemental Figure 3C). The exchange of threonine to proline redirects the dihedral angles Φ and Ψ away from the range favored for β-strand formation, shortening the β-strand (aa 541–549) by 2 amino acids (Supplemental Figure 3D). In addition, the polar contacts between T542 and F603 of the adjacent antiparallel β-strand are disrupted, destabilizing this β-sheet and potentially compromising β-strand mediated protein-protein interaction via an adjacent β-sheet of FANCB in the BLP100 module.

### FAAP100^T542P^-mutant 1176 cells are sensitive to ICL induction, which is complemented by FAAP100^WT^.

**Figure 2:**
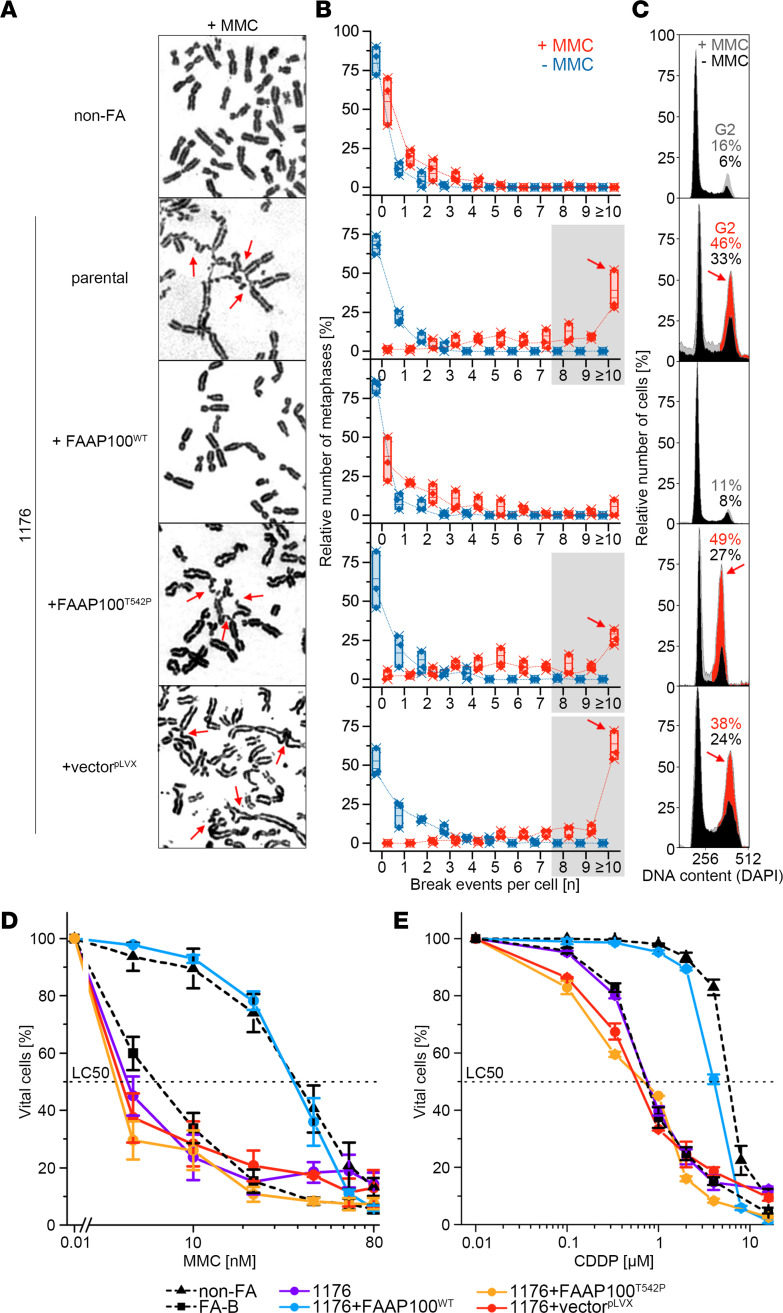
Ectopic expression of FAAP100^WT^ or FAAP100^T542P^ in FAAP100-deficient cells. (**A**) Metaphase micrographs of FAAP100^T542P^-mutant 1176 cells after exposure of the cultures to MMC (100 ng/mL, 48 hours). Parental 1176 cells and mock- or mutation-transduced (+vector^pLVX^ or +FAAP100^T542P^) 1176 cells show distinctly increased chromosome breakage, mostly of the chromatid type, whereas WT transduced 1176+FAAP100^WT^ cells are rescued. Radials are marked with red arrows. (**B**) Box plots reflect the proportion of cells with the indicated number of chromosome breaks per metaphase; single value (♦), median (**─**), mean (**□**), IQR (**─**), minimum (×), and maximum (×) for the number of breaks from 3 independent experiments; blue symbols are from untreated cultures, and red are from cultures exposed to MMC (100 ng/mL, 48 hours). Light gray shading indicates high rates of 8 or more breaks per metaphase, and red arrows highlight pivotal rates of 10 or higher. Cell lines are the same as in **A**. Fifty metaphases were analyzed per experiment. (**C**) Cell-cycle analysis by flow cytometry. Exemplary individual measurements. Black histograms are from untreated cultures, and superimposed gray or gray/red are from cultures exposed to MMC (10 ng/mL, 48 hours). The percentage of cells in the G_2_ phase is indicated. Red coloring and arrows denote an increased G_2_ compartment size. Variability and significance of G_2_ phase arrest in repeated measurements are shown in Supplemental Figure 4A. Cell lines are the same as in **A**. (**D** and **E**) Dose-response (survival) curves of parental and mock FAAP100^WT^ or FAAP100^T542P^ transduced 1176 cells from cultures exposed to different concentrations of MMC (**D**) or CDDP (**E**) for 8 days. Data indicate the mean ± SD of 3 independent experiments. Cell lines are the same as in **A** and are identical in **D** and **E**. FA-B and non-FA are FA and normal control, respectively. LC_50_, 50% lethal concentration. Note that the transduction of FAAP100^WT^ complements the repair defect in all assays, whereas FAAP100^T542P^ does not.

**Figure 3:**
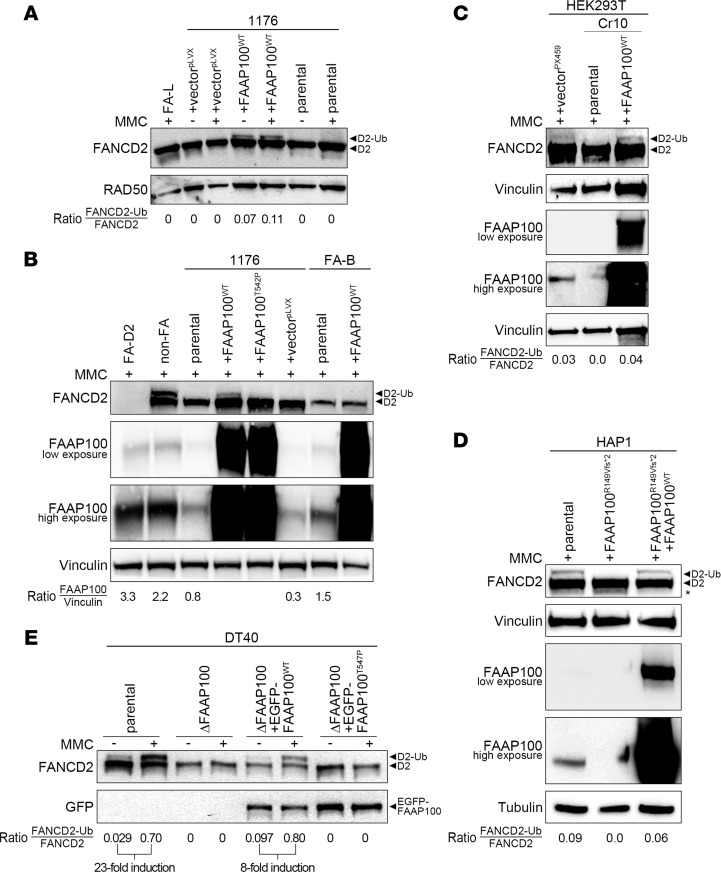
FANCD2 monoubiquitylation capacity of FAAP100-inactivated human and avian cell lines. (**A**) FAAP100^T542P^ mutant 1176 cells (parental) lacked FANCD2 monoubiquitylation (◄D2) on immunoblots. FANCD2 monoubiquitylation was restored only in 1176+FAAP100^WT^ transduced cells (◄D2-Ub), but not in 1176+FAAP100^T542P^ or mock transduced cells. FA-L was a monoubiquitylation-deficient FA control. (**B**) FAAP100^T542-^mutant 1176 cells showed reduced levels of FAAP100 protein (see ratio to vinculin in parental and +[empty] vector^pLVX^ lanes) and lacked FANCD2 monoubiquitylation (◄D2) on immunoblots. 1176+FAAP100^WT^- and 1176+FAAP100^T542P^-transduced cells both overexpressed FAAP100, but FANCD2 monoubiquitylation was restored only in 1176+FAAP100^WT^ cells (◄D2-Ub). FA-D2 was a control for absent FANCD2, and non-FA was a monoubiquitylation-proficient normal control. (**C**) FANCD2 monoubiquitylation was functional in HEK293T cells (not shown) and HEK293T cells mock transfected with the Cas9-containing vector PX459 (◄D2-Ub), but not in the FAAP100-inactivated HEK293T CRISPR/Cas9 clone Cr10 (parental) (◄D2), where it was rescued by transduction with FAAP100^WT^, resulting in overexpression of FAAP100 (◄D2-Ub), as shown by FANCD2 and FAAP100 immunoblots. (**D**) FANCD2 monoubiquitylation was functional in parental HAP1 cells (◄D2-Ub), but not in FAAP100-inactivated HAP1 FAAP100^R149Vfs*2^ cells (◄D2), where it was restored by transduction with FAAP100^WT^, resulting in overexpression of FAAP100 (◄D2-Ub), as shown by FANCD2 and FAAP100 immunoblots. (**E**) FANCD2 monoubiquitylation was functional in parental DT40 cells (◄D2-Ub), but absent in ΔFAAP100-DT40 cells (◄D2). In the latter, it was rescued by the expression of EGFP-FAAP100^WT^ (◄D2-Ub), but not by EGFP-FAAP100^T547P^ (◄D2), which contained the chicken equivalent T547P of human T542P, as shown by FANCD2 and EGFP immunoblots. Note that the strong overexpression of the FAAP100 protein by lentiviral transduction in **B**–**D**, where low- and high-exposure blots are shown, was in marked contrast to the transfection in **E**. Vinculin or RAD50 or tubulin was used as a loading control on all blots. The FAAP100/vinculin ratios were taken from the high-exposure FAAP100 blot. The FANCD2-Ub/FANCD2 ratios semiquantitatively estimated monoubiquitylation.

To experimentally test the potential pathogenicity of the FAAP100^T542P^ variant, cultured 1176 fibroblasts were transduced with *FAAP100^WT^*, *FAAP100^T542P^*, and empty vector pLVX as a sham control. The response of the cells to ICL-inducing chemical agents was assessed by various techniques. Chromosome breakage analysis revealed the accumulation of metaphases with high breakage rates (≥8 breaks per nucleus), including frequent radial figures, after exposure to MMC of nontransduced (parental) 1176 cells, 1176 cells that were transduced with *FAAP100^T542P^*, or mock-transduced 1176 cells (Figure 2, A and B). In contrast, 1176 cells ectopically expressing transduced *FAAP100^WT^* showed a break number distribution similar to non-FA control fibroblasts and very few metaphases with high break rates. In cell-cycle studies, the FA-typical G2 phase accumulation in parental 1176 cells in response to MMC exposure was restored to normal control levels in *FAAP100^WT^*-transduced 1176 cells, but not in *FAAP100*^T542P^ transduced or mock-transduced 1176 cells (Figure 2C; for variability, compare Supplemental Figure 4A). Furthermore, survival after exposure to MMC or cisplatin (CDDP) was rescued in *FAAP100^WT^*-transduced 1176 cells, but not in parental, *FAAP100^T542P^*-transduced or mock-transduced 1176 cells, or in FA-B fibroblasts (Figure 2, D and E). A consequence of the homozygous c.1624A>C (FAAP100^T542P^) mutation in 1176 cells was defective FANCD2 monoubiquitylation (Supplemental Figure 5A), whereas the monoallelic c.1624A>C mutation status in cells from the unaffected heterozygous parents was associated with sufficient FANCD2 monoubiquitylation (Supplemental Figure 5B), suggesting that the FAAP100 mutation was nonfunctional in a recessive manner. Transduction of *FAAP100^WT^* complemented deficient FANCD2 monoubiquitylation in 1176 cells (Figure 3A), whereas transduction of *FAAP100^T542P^* did not (Figure 3B). Transduction of *FAAP100^WT^* or *FAAP100^T542P^* did not alter the deficient FANCD2 monoubiquitylation status of FA-B cells or the proficient FANCD2 monoubiquitylation of non-FA cells (Supplemental Figure 5, C and D). Furthermore, parental 1176 cells and, similarly, 1176 cells transduced with *FAAP100^T542P^* or empty vector were deficient in the formation of FANCD2 subnuclear foci, an accumulation of monoubiquitylated FANCD2 and other DNA repair proteins on chromatin (Supplemental Figure 6A). Transduction of *FAAP100^WT^* rescued the defect in 1176 cells (Supplemental Figure 6, A and B), whereas we observed no significant change in FANCD2 foci formation by the transduction of either type of *FAAP100* in FA-B fibroblasts (deficient) or non-FA normal control fibroblasts (proficient) (Supplemental Figure 6C). Deficient FANCD2 monoubiquitylation was also the effect of FAAP100 silencing by depletion through transfection of HeLa cells with *FAAP100* siRNA (Supplemental Figure 7, A and B).

### FAAP100-targeted established human and avian cell lines are sensitive to ICL induction and are complemented by FAAP100^WT^.

We also studied the effects of FAAP100 deficiency in established cell lines genetically engineered to homozygously disrupt the *FAAP100* gene. These lines included the CRISPR/Cas9 *FAAP100* edited clones Cr10 and Cr12 derived from HEK293T cells, FAAP100^R149Vfs*^ cells derived from HAP1 cells, and ΔFAAP100-DT40 cells derived from DT40 cells by chicken *FAAP100* targeting (23). HEK293T Cr10 and HAP1-FAAP100^R149Vfs*^ cells have a small duplication and deletion, respectively, resulting in frameshifts and truncation of the FAAP100 protein (Supplemental Table 3 and Supplemental Figure 8A). HEK293T Cr12 cells have an internal 27 bp in-frame *FAAP100* deletion, and ΔFAAP100-DT40 cells have a large genomic *FAAP100* deletion (Supplemental Table 3 and Supplemental Figure 8A). FAAP100 protein was not detected in HEK293T Cr10, HAP1-FAAP100^R149Vfs*2^ (Figure 3, C and D, and Supplemental Figure 8B), or ΔFAAP100-DT40 cells (23), in contrast to the presence of residual mutant FAAP100 protein in Cr12 cells (Supplemental Figure 8, B and C). HEK293T Cr12 cells also showed a residual mutant FAAP100 protein levels that were determined by titration to be approximately 1/27th of normal levels, similar to 1176 cells (Supplemental Figure 8C); a theoretical, small size difference compared with FAAP100^WT^ could not be reliably detected (Supplemental Table 3 and Supplemental Figure 8B). HEK293T Cr10, HEK293T Cr12, and HAP1-FAAP100^R149Vfs*2^ cells overexpressed the full-length protein after transduction with *FAAP100^WT^* or *FAAP100^T542P^*, as did 1176 cells (Figure 3, B–D), whereas expression, but not overexpression, of FAAP100 was observed after transfection of ΔFAAP100-DT40 cells (Figure 3E). The *FAAP100*-targeted cell lines exhibited MMC or CDDP sensitivity with FA-typical characteristics very similar to those of 1176 cells and, like 1176 cells, were rescued by the transduction with *FAAP100^WT^* but not *FAAP100^T542P^* or, in the case of ΔFAAP100-DT40 cells*,* by transfection of *chFAAP100^WT^* but not *chFAAP100^T547P^*, with the latter containing the chicken equivalent T547P of human T542P (Supplemental Figures 9–11; for G_2_ phase variability, compare Supplemental Figure 4, B and C). Transduction of FANCD2 monoubiquitylation–deficient HEK293T Cr10 or HAP1-FAAP100^R149Vfs*2^ cells with *FAAP100^WT^* rescued FANCD2 monoubiquitylation (Figure 3, C and D). Similarly, MMC-sensitive ΔFAAP100-DT40 cells were deficient in FANCD2 monoubiquitylation (Figure 3E). After stable transfection, they expressed EGFP- or FLAG-chFAAP100^WT^ protein, or EGFP- or FLAG-chFAAP100^T547P^ protein (Figure 3D). Only EGFP- or FLAG-chFAAP100^WT^, but not EGFP- or FLAG-chFAAP100^T547P^, rescued FANCD2 monoubiquitylation and survival (Supplemental Figure 11 and Figure 3E). These results underscore the idea that functional FAAP100 is an absolute requirement for the repair of ICL-like DNA defects and that FAAP100^T542P^ or chFAAP100^T547P^ are nonfunctional in this regard.

### faap100 KO in zebrafish results in FA phenotypes.

**Figure 4:**
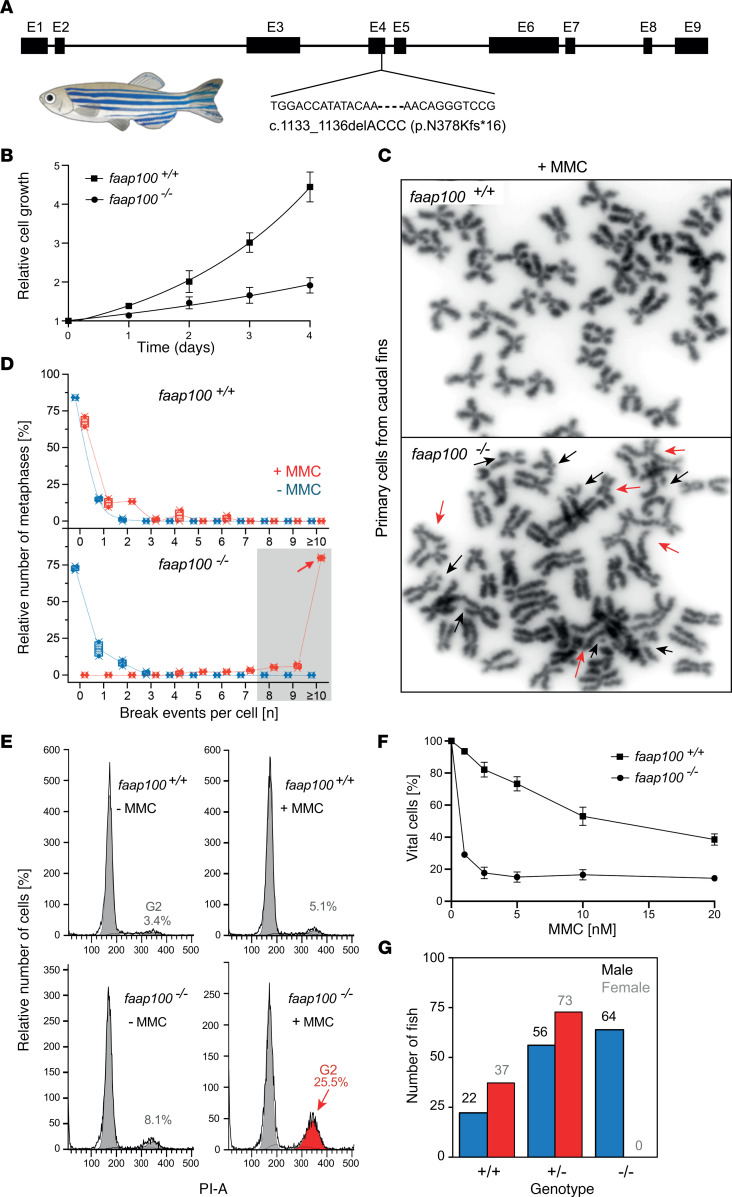
Cellular FA phenotype of *faap100*-KO zebrafish. (**A**) Schematic of the CRISPR/Cas9-mediated *faap100* gene–KO (si:dkey-57h18.1). (**B**) Growth curves show slower proliferation of primary cell cultures from *faap100^–/–^* fish (circles) than from *faap100^+/+^* fish (squares). The mean ± SD for multiples of the initial cell number of 3 independent subcultures is shown for each time point, with day 0 counts set to 1. ***P < 0.001 (*t* test) at day 4. Data were exponentially fitted. (**C**) Metaphase micrographs after exposure of *faap100^+/+^* or *faap100^–/–^* cell cultures to MMC (2.5 ng/mL, 24 hours). *faap100^–/–^* cells showed markedly increased chromosome breakage, mostly of the chromatid type. Red arrows indicate radials and black arrows other types of breakage. Original magnification, ×1,000. (**D**) Box plots reflect the proportion of *faap100^+/+^* (top) or *faap100^–/–^* (bottom) cells with the indicated number of chromosome breaks per metaphase. Single value (♦), median (**─**), mean (**□**), IQR (**─**), minimum (x), and maximum (x) for the number of breaks from 2 independent experiments; blue symbols represent data from untreated cultures, and red symbols represent data from cultures exposed to MMC (2.5 ng/mL, 24 hours). Light gray shading indicates high rates of 8 or more breaks per metaphase, and the red arrow highlights a pivotal rate of 10 or higher. A total of 31–53 metaphases were analyzed per experiment. (**E**) Flow cytometric cell-cycle analysis of *faap100^+/+^* (top) and *faap100^–/–^* (bottom) cell cultures without MMC (– MMC) or after exposure to MMC (+ MMC) (5 ng/mL, 48 hours). Exemplary individual measurements are shown. The percentages of cells in G_2_ are shown. Red coloring and arrow indicate an increased G_2_ compartment size. (**F**) Dose-response (survival) curves of *faap100^+/+^* (top) and *faap100^–/–^* (bottom) cells from cultures exposed to different concentrations of MMC. The mean ± SD of triplicates is shown. (**G**) Homozygous KO of *faap100* resulted in complete sex reversal from female to male. The numbers above the bars represent the number of fish in each sex and genotype category.

We further investigated the consequences of faap100 deficiency in a previously generated zebrafish *faap100*-KO (ENSDARG00000079457) model using CRISPR/Cas9-mediated gene editing (27). Here,we report the functional characterization of primary cell cultures established from caudal fins of *faap100^–/–^* fish homozygous for the *faap100* c.1133_1136delACCC, Δ4 (hg72) deletion (Figure 4A). We performed cell growth studies and chromosome breakage, cell-cycle, and survival analyses. *faap100^–/–^* cells in culture showed significantly reduced growth compared with WT cultures (Figure 4B). The doubling time of *faap100^–/–^* cultures was almost 3 times slower than that of WT controls (5.13 vs. 1.81 days). Chromosome breakage analysis of *faap100^–/–^* cells after exposure to MMC revealed the accumulation of metaphases with high breakage rates (≥8 breaks per nucleus), including multiple radial figures (Figure 4, C and D). Cell-cycle analysis showed the FA-typical disproportionately increased G_2_-phase arrest following exposure to MMC (Figure 4E). In addition, decreased *faap100^–/–^* cell survival was observed after exposure to MMC (Figure 4F). These results demonstrate that zebrafish cells required faap100 to prevent MMC-induced chromosomal and cell-cycle aberrations and cell death. Furthermore, homozygous mutants of 17 zebrafish *fanc* genes had displayed a female-to-male sex reversal phenotype (27), highlighting the role of FA pathway genes in meiotic oocyte survival. Here, sex determination of adult fish revealed that *faap100^–/–^* zebrafish also had complete female-to-male sex reversal (Figure 4G). We tested the fertility of male homozygous KOs by outcrossing them with WT fish and found no fertility defects (data not shown), which is consistent with the observations for most (15 of 17) of the zebrafish *fanc* gene KOs (27). In summary, our zebrafish data show that disruption of *faap100* resulted in functional cellular phenotypes consistent with *fanc* gene KOs.

### A customized Faap100-KO mouse recapitulates the phenotypes observed in other FA mouse models.

**Figure 5:**
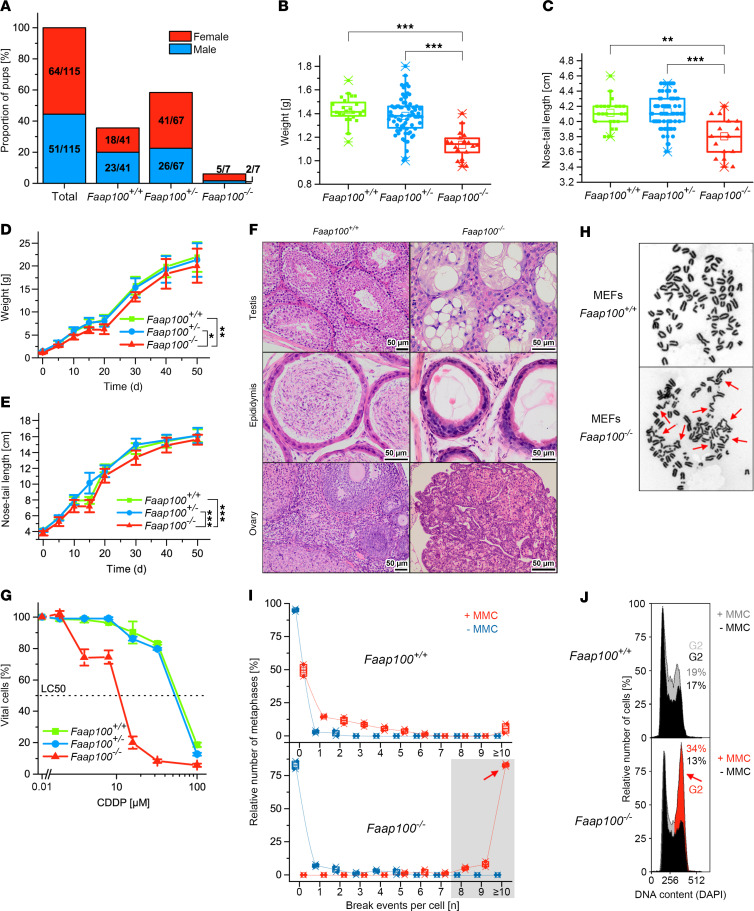
Characteristics of the *Faap100^–/–^* mouse. (**A**) Proportions and numbers of female (red) and male (blue) *Faap100^+/+^*, *Faap100^+/–^*, and *Faap100^–/–^* mouse offspring from heterozygous mating. (**B**) *Faap100^–/–^* mice show significantly lower birth weight. Box-and-whisker plots: single value (■, ●, **▲**), median (─), mean (□), IQR (─), whiskers (–), and range (x). *n* = 24 (*Faap100^+/+^*), *n* = 73 (*Faap100^+/–^*), and *n* = 18 (*Faap100^–/–^*). ***P < 0.001, by 1-way, repeated-measures ANOVA with Tukey’s test (**B** and **C**). (**C**) Shortened nose-to-tail length in *Faap100^–/–^* neonatal mice. *n* = 23 (*Faap100^+/+^*) , *n* = 68 (*Faap100^+/–^*), and *n* = 15 (*Faap100^–/–^*). **P 0.01 and ***P < 0.001. (**D**) Reduced postnatal weight gain in *Faap100^–/–^* mice. *n* = 9–30 (*Faap100^+/+^*), *n* = 10–83 (*Faap100^+/–^*), and *n* = 6–11 (*Faap100^–/–^*). *P < 0.05 and **P < 0.01, by 2*-*way, repeated-measures ANOVA with post hoc Tukey’s test (**D** and **E**). (**E**) Slower growth in body length of *Faap100^–/–^* mice. *n* = 9 (*Faap100^+/+^*), *n* = 11–42 (*Faap100^+/–^*), and *n* = 9 (*Faap100^–/–^*). ***P < 0.001. (**F**) Gonads in *Faap100^–/–^* mice appear dysplastic, unlike normal gonads in *Faap100^+/+^* mice. Scale bars: 50 μm. (**G**) Dose-response survival curves of MEFs exposed to CDDP (8 days) show reduced survival of *Faap100^–/–^* cells. Data are the mean ± SD of triplicates. LC_50_, 50% lethal concentration. (**H**) Metaphase micrographs after MMC exposure (100 ng/mL, 48 hours) show increased radials (red arrows) and chromatid breaks in *Faap100^–/–^* MEFs. Original micrographs have been magnified approximately ×1,000. (**I**) Box plots of breaks per metaphase from 2 independent experiments. Red symbols: MMC-treated; blue: untreated. Gray zone = 8 or more breaks; red arrow = 10 or more breaks. *n* = 50 metaphases/experiment. (**J**) Cell-cycle analysis reveals G_2_-phase arrest in *Faap100^–/–^* MEFs after MMC exposure (10 ng/mL, 48 hours). Exemplary individual measurements. Black histograms: untreated; gray or gray/red overlaid histograms: MMC-treated. The percentage of G_2_ cells is indicated. Red coloring and arrow: increased G_2_ compartment size. Variability and significance of G_2_-phase arrest in repeated measurements are shown in Supplemental Figure 4D.

As another animal model of FAAP100 deficiency, we generated and studied an *Faap100*-KO mouse. Cre-induced deletion of exon 3 resulted in the mutation c.285_1240del with the deduced effect p.(Ser96*) (Supplemental Figure 12A). Male goGermline mice were bred with C57BL/6 females to produce heterozygous *Faap100^+/–^* germline offspring on a C57BL/6 background (28). Gene dosage studies identified *Faap100^+/+^*, *Faap100^+/–^*, and *Faap100^–/–^* mice (Supplemental Figure 12B). Real-time quantitative PCR (RT-qPCR) analysis of normalized relative *Faap100* mRNA expression in *Faap100^+/+^* and *Faap100^–/–^* mice revealed virtually no amplification of a transcript region bridging exons 3–4 in *Faap100^–/–^* mice (Supplemental Figure 12C). This confirmed the genomic deletion of exon 3 at the mRNA level. Notably, *Faap100* mRNA spanning exons 6–8 was present in *Faap100^–/–^* mice at approximately half the level of *Faap100^+/+^* mice (Supplemental Figure 12D). This indicates the expression of *Faap100* transcripts that, however, lacked the sequence associated with exon 3. This skipping shifted the downstream exons out of the *Faap100* reading frame and rendered these transcripts nonfunctional. *Faap100^–/–^* mice were viable but were born at a sub-Mendelian rate of 6.1% offspring from heterozygous mating, suggesting embryonic and/or fetal lethality (Figure 5A). *Faap100^–/–^* pups had a significantly reduced birth weight and nose-to-tail length (Figure 5, B and C). *Faap100^–/–^* mice also showed a small but significant impairment in weight gain and nose-to-tail growth (Figure 5, D and E). Histological examination of *Faap100^–/–^* mice revealed testicular and ovarian atrophy (Figure 5F). In *Faap100^–/–^* males, the testes showed atrophic seminiferous tubules. Most appeared empty, with little evidence of any active cell division. Spermatozoa were absent from the epididymis. Female *Faap100^–/–^* mice had ovarian hypoplasia with small, malformed ovaries and very immature and dysfunctional ovarian tissue, with a predominance of stromal and luteal cells but inactive epithelium. No follicular differentiation or development was observed. The oviducts were relatively well differentiated. These findings suggest that sexual differentiation was largely intact in *Faap100^–/–^* mice, but germ cell formation was abolished. *Faap100^–/–^* mice were consistently sterile in various test mating constellations. Anophthalmia, hydrocephalus, and limb malformations were observed in 6 of 62 *Faap100^–/–^* and *Faap100^+/–^* mice (Supplemental Table 4). The rate of malformations was slightly higher than that published for basic anomalies in newborn, inbred C57BL/6 mice (29). In addition, 23 *Faap100^+/+^* mice maintained under the same conditions showed no malformations. Pancytopenia or bone marrow failure, as assessed by peripheral blood counts, was not present in *Faap100^–/–^* mice in a 6-month pilot study (Supplemental Table 5). *Faap100^–/–^* mouse embryonic fibroblasts (MEFs) were obtained from embryonic tissue cultures and confirmed for the absence or heterozygous or homozygous presence of the *Faap100* exon 3 deletion (Supplemental Figure 12E). *Faap100^–/–^* MEFs exhibited decreased survival after exposure to CDDP (Figure 5G). Karyotypes showed frequent radial-like chromatid exchanges, increased breakage predominantly of the chromatid type, and an accumulation of metaphases with a high number of breaks (≥8 breaks per nucleus) after exposure to MMC (Figure 5, H and I). *Faap100^–/–^* MEFs also showed increased MMC-induced G_2_-phase arrest (Figure 5J; for G_2_ phase variability, compare Supplemental Figure 4D). Taken together, the organismic and cellular characteristics of *Faap100^–/–^* mice appeared compatible with an FA phenotype.

### The FAAP100^T542P^-mutant protein disrupts FA core complex functions biochemically and mechanistically.

**Figure 6:**
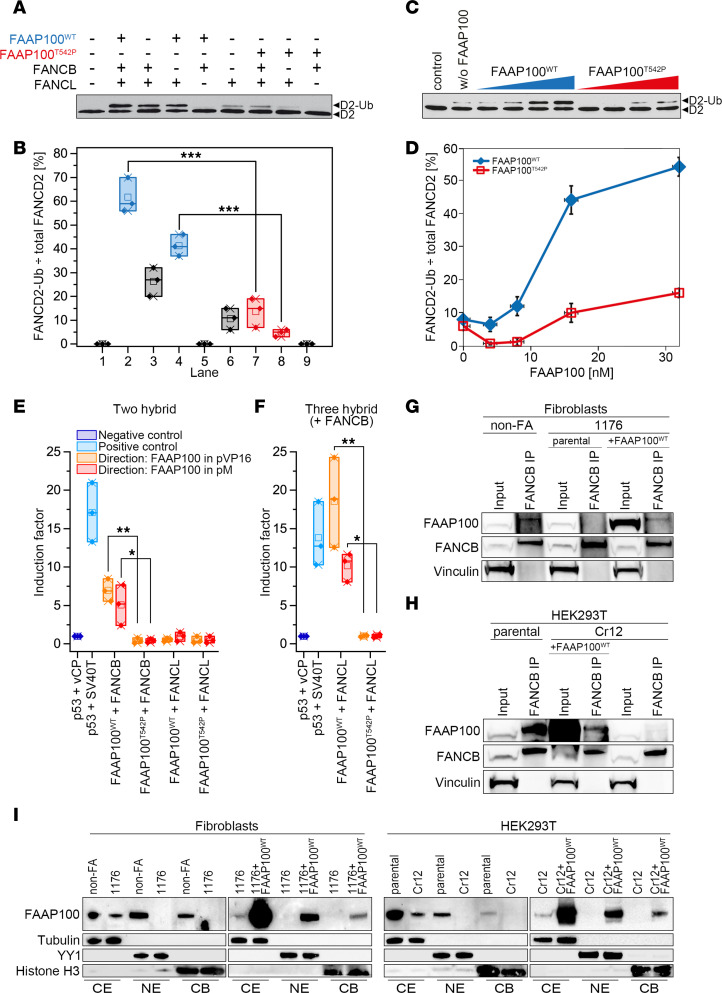
Ligase activity, interaction, and subcellular localization studies with FAAP100^T542P^. (**A**) Reconstitution of FANCD2 monoubiquitylation using purified proteins, including HA-ubiquitin, UBE1, UBE2T, and FANCD2-FANCI complex and, as indicated, FAAP100^WT^ or FAAP100^T542P^, FANCB, and/or FANCL. FANCD2 immunoblot: ◄D2-Ub, monoubiquitylated; ◄D2, nonubiquitylated. (**B**) Quantitation of results from **A**. FANCD2-Ub divided by total FANCD2 (percentage) indicates the ubiquitylation efficiency. Lane numbers are identical to those in **A**. Box plots: single value (**♦**), median (─), mean (□), IQR (─), minimum (x), and maximum (x) from 3 independent experiments. ***P < 0.001, by 1-way, repeated-measures ANOVA with post hoc Tukey’s test. (**C**) Titration of FAAP100^WT^ (blue) and FAAP100^T542P^ (red). For reaction mixtures, see **A**, including FAAP100^WT^ or FAAP100^T542P^, FANCB, and FANCL. (**D**) Quantitation of results from **C** (see **B** and **C** for details). Data indicate the mean ± SD of 3 independent experiments. (**E**) In mammalian 2-hybrid assays, FAAP100^WT^, but not FAAP100^T542P^, interacted with FANCB. FAAP100 fused to the activation domain (orange) or the DNA-binding domain (red). Neither direction of FAAP100 fusion directly interacted with FANCL. Box plots: single value (♦), median (─), mean (**□**), IQR (─), minimum (x), and maximum (x) of 3 independent experiments. Induction factor, multiples of negative control. *P < 0.05 and **P < 0.01, by 1-way, repeated-measures ANOVA with post hoc Tukey’s test. (**F**) In mammalian 3-hybrid assays, FAAP100^WT^, but not FAAP100^T542P^, interacts with FANCL in both fusion directions in the presence of stably overexpressed FANCB. Controls, calculations, and statistical tests are the same as in **E**. *P < 0.05 and **P < 0.01, by 1-way, repeated-measures ANOVA with post hoc Tukey’s test. (**G** and **H**) Co-IPs from cells transfected with FANCB vector and exposed to MMC (40 ng/mL,16 hours). FAAP100^T542P^ in 1176 cells and FAAP100^L543_S551del^ in HEK293T clone Cr12 did not interact with FANCB. Transduced FAAP100^WT^ rescued the pull-down of FAAP100 by FANCB. Vinculin was used as a loading control. (**I**) On subcellular protein fractionation, FAAP100^T542P^ in 1176 fibroblasts and FAAP100^L543_S551del^ in the HEK293T clone Cr12 were not detected in nuclear extracts (NE) or on chromatin (CB). Transduced FAAP100^WT^ rescuedFAAP100 relocalization. CE, cytoplasmic extracts. Tubulin, YY1, and histone H3 were used as loading controls. Cells were exposed to MMC (40 ng/mL, 16 hours).

To determine whether FAAP100^T542P^ affects FANCD2 monoubiquitylation in a biochemically defined system, we reconstituted the FANCD2 monoubiquitylation reaction in vitro using purified human FANCD2-FANCI (30), FAAP100^WT^ (Supplemental Figure 13A), FAAP100^T542P^, FANCB, and FANCL as indicated in Figure 6A, in the presence of purified ubiquitin (UB), ubiquitin-activating enzyme E1 (UBE1), and ubiquitin-conjugating enzyme E2 T (UBE2T) (31). With FANCL alone, only limited monoubiquitylation of FANCD2 occurred (Figure 6B, lane 6). As described previously (31), FAAP100^WT^ dramatically enhanced FANCL-mediated monoubiquitylation of FANCD2 (Figure 6B, compare lanes 2 and 4 with lane 6). In contrast, FAAP100^T542P^ was defective in stimulating the FANCD2 monoubiquitylation reaction in the presence of FANCB (Figure 6B, compare lane 7 with lane 2, and Figure 6C). Similarly, the FAAP100^T542P^ mutant was also significantly less efficient than FAAP100^WT^ in the absence of FANCB (Figure 6B, compare lane 8 with lane 4, and Figure 6C). In conclusion, FAAP100^T542P^ was defective in stimulating FANCL-mediated monoubiquitylation of FANCD2. A titration experiment, performed to exclude potential inhibitory or dominant-negative effects, further confirmed the stimulatory defect of FAAP100^T542P^ (Figure 6, C and D). In support of this observation, we also found that, unlike FAAP100^WT^, FAAP100^T542P^ was unable to form a complex with FANCB and FANCL in insect cells. As shown in Supplemental Figure 13B, cotransfection of FANCB, FANCL, and FAAP100^WT^ baculoviruses in insect cells resulted in the formation of the BLP100 complex, which could be purified as a trimeric entity (Supplemental Figure 13B, lanes 2, 4, and 6). However, purification of a putative complex after coexpression of FANCB, FANCL, and FAAP100^T542P^ by baculovirus in insect cells yielded only a purified FANCB protein without FANCL and FAAP100 (Supplemental Figure 13B, lanes 3, 5, and 7). To further investigate the interaction of FAAP100^T542P^ with FANCB and FANCL in a different cellular background, we performed mammalian 2- and 3-hybrid (M2/3H) assays in HEK293T cells. M2H studies revealed no interaction between FAAP100^T542P^ and FANCB (Figure 6E). Consequently, M3H experiments showed no interaction between FAAP100^T542P^ and FANCL in the presence of FANCB (Figure 6F). Co-IP studies in 1176 cells using FANCB for pull-down confirmed the absence of FAAP100^T542P^ binding to FANCB (Figure 6G). A similar result was obtained in HEK293T Cr12 cells expressing the FAAP100^L543_S551del^ CRISPR mutant (Figure 6H). The BLP100 subcomplex is normally formed in the cytosol and imported into the nucleus in a FANCA- and FANCM-dependent manner (13, 23). Our in silico analysis confirmed that FAAP100 did not possess a core nuclear localization signal (NLS). Subcellular protein fractionation of 1176 and HEK293T Cr12 cells revealed that, in contrast to FAAP100^WT^, FAAP100^T542P^ or FAAP100^L543_S551del^ was present only in the cytoplasm and was unable to enter the nucleus and access chromatin (Figure 6I). In summary, the mutagenic effect of FAAP100^T542P^ was manifested at several levels: it failed to support the E3 ubiquitin ligase activity of FANCL toward FANCD2 because it abolished the normal interaction of FAAP100 with FANCB so that no BLP100 complex was formed, and because of the lack of indirect interaction of FAAP100 with FANCA, no nuclear translocation occurred.

## Discussion

Here, we propose the identification of *FAAP100* as the 23rd *FANC* gene, FAAP100 alias *FANCX*. Several lines of evidence support this suggestion. Starting with a fetus with malformations suggestive of FA that was not carried to term, we identified a homozygous mutation in fetal cells, *FAAP100* c.1624A>C, resulting in FAAP100^T542P^. It segregated in the consanguineous core family, consistent with an autosomal recessive trait and similar to most types of *FANC* gene mutations (4). The presence of the mutation was associated with hypersensitivity to ICL induction in fetal 1176 cells. This effect was reproduced in human HEK293T and HAP1 cells, as well as in chicken, zebrafish, and mouse cells after mutagenesis in *FAAP100*, indicating that FAAP100 triggers ICL repair across vertebrate species. Complementation with WT *FAAP100* rescued all cell types. Consistent with FAAP100 being a constitutive component of the FA core complex, the pathogenic variant abolished FANCD2 monoubiquitylation and FANCD2 foci formation. These findings were consistent with previous results obtained with *FAAP100*-depleted HeLa or avian KO cells (23). Biochemically, FAAP100^T542P^ fails to enhance the reported ubiquitin ligase activity of FANCL in vitro (30, 31). Our observation that FAAP100^T542P^ abrogated BLP100 subcomplex formation and FAAP100 translocation to chromatin is consistent with observations that FAAP100 confers stability to the BLP100 subcomplex, which relocalizes as a whole to the nucleus in a FANCA-dependent manner (23).

The human *FAAP100* gene has 1 canonical transcript and predicted or theoretical alternative transcripts, only a subset of which are potentially coding and classified at Transcript Support Level 1 in Ensembl (https://www.ensembl.org/). Just one has been validated, ENST00000443656.6, FAAP100-203, NR_033338.1, which is subject to nonsense-mediated RNA decay. Analysis of sample publicly available RNA-Seq data confirmed 3 alternative transcripts (https://isomix.org/isovis/), with FAAP100-202 and FAAP100-203 being listed in Ensembl (https://www.ensembl.org/) (Supplemental Figure 1D). Of the 2 alternative FAAP100 transcripts examined in cell line 1176, one was identical to FAAP100-203. All alternative transcripts analyzed represented RNA isoforms due to alternative splicing, and all contained the variant cDNA position c.1624A>C (Supplemental Figure 1D), providing no evidence for the possibility of mutation skipping. Our extended investigation of a previously generated zebrafish *faap100^–/–^* model from a study systematically targeting the FA pathway in zebrafish (27) further supports the classification of *faap100* as a *fanc* gene. *FAAP100* has only 1 ortholog in zebrafish for the human gene (32). This puts *faap100* in line with the *fanc* genes, all of which quickly reverted to singletons after whole-genome duplication during teleost fish evolution, possibly by dosage selection for stoichiometric reasons, while their products often act in dosage-sensitive complexes, pathways, or networks (33). The *faap100^–/–^* zebrafish show a complete female-to-male sex reversal, consistent with 17 other zebrafish *fanc* gene–KO models (27). Zebrafish sex is primarily determined by the number of meiotic oocytes in the juvenile ovary, but when the number of meiotic oocytes is reduced by p53-activated apoptosis during the early stages of development driven by FA genotypes, this results in testis development (34). Beyond sex reversal, there was no phenotypic presentation of *faap100^–/–^* zebrafish similar to 17 zebrafish *fanc* and the 2 *faap100* and *faap20* gene–KO models (27). The reasons that single or double *fanc* gene–KO models do not exhibit malformations or develop neoplasms are not entirely clear, even though zebrafish lack the *brca1* gene (32). Persistence of maternal RNA and prolonged maternal control of early zebrafish development may be one possible reason for the absence of gross developmental abnormalities in FA zebrafish models (33), facilitated by 5-methylcytosine (m^5^C) modification of maternal RNA in early zebrafish embryos, which confers high RNA stability (35). In contrast, we found strong support for *faap100^–/–^* as a *fanc* gene at the cellular level, consistent with the ICL hypersensitivity described in zebrafish KO models of the other genes encoding proteins of the FA nuclear core complex (27).

To analyze *Faap100* functions in a mammalian model system, we generated a new customized *Faap100*-KO mouse, although we recognize that FA mouse models only partially recapitulate human FA disease (36). Viable *Faap100^–/–^* mice were born at a sub-Mendelian ratio, consistent with some FA mouse models (36), suggesting prenatal loss that occurs primarily on the C57BL/6J background by a mechanism not fully explored in this or other FA mouse models. The small litter size of *Faap100^–/–^* pups and short body lengths and slow weight gain of *Faap100^–/–^* mice are consistent with an FA phenotype in a subset of FA mouse models, as in humans. Although a full characterization will require future studies, we observed individual malformations in *Faap100^–/–^* mice at a cumulative rate just above that expected for spontaneous malformations in C57BL/6J mice (29). Most other FA mouse models also lack the gross developmental abnormalities characteristic of human FA disease (36). Recurring traits include embryonic and perinatal mortality, growth retardation, and microphthalmia in some FA mice, and hypogonadism and subfertility in most, if not all, FA mouse models. The latter has been attributed to the attrition of primordial germ cells during their expansion phase, mediated by accumulated DNA damage, G_2_ phase arrest, and p-p53–mediated apoptosis (24, 36). Consistently, the prominent feature of the present *Faap100^–/–^* mouse model was gonadal atrophy with corresponding infertility. The combination of the sub-Mendelian ratio of only 6.1% of *Faap100^–/–^* pups at birth, the presence of developmental abnormalities, and severe gonadal atrophy makes the *Faap100^–/–^* mouse one of the more prominent FA mouse models, based on information drawn from several reviews (36–38). The observation period in our study was too short to systematically evaluate the *Faap100^–/–^* mouse model in terms of bone marrow failure (BMF) or neoplasia. Double-KOs including Faap100 and the combination of exemplar mutations of different *Fanc* and other genes hold the promise to do both, revealing mutational synergisms and more faithfully modeling FA disease (39).

With no clear evolutionary record and recognizable homologs only in vertebrates, *FAAP100* is what might be called an orphan gene (40). The lack of recognized functional motifs, domains, or enzymatic activity did not predict putative protein functions. Nevertheless, homology searches proved instructive regarding its evolutionary history. FAAP100 shares homology with a conserved protein family termed FANCAA (Pfam protein families database; http://pfam.xfam.org/; https://www.ebi.ac.uk/interpro/), which is widely represented in FAAP100 of all vertebrate species (Supplemental Figure 14, A and B). In human FAAP100, the region occupied by FANCAA comprises the 432 C-terminal amino acids including the residue T542, which is mutated in our present study (Supplemental Figure 14C). The conservation of this and the adjacent amino acids suggests that the T542P substitution not only affects the functions of FAAP100 but also interferes with the contiguity of FANCAA. The FANCAA element in FAAP100 is very similar in size and C-terminal position across vertebrate species (Supplemental Figure 14C). In invertebrates and even some plants and prokaryotes, we have identified portions of the FAAP100 FANCAA element by forward and reverse searches in a few proteins that can be recognized as FAAP100-like and in other proteins that are unrelated or described as uncharacterized, predicted, or hypothetical. These segments are conserved for both sequence and structural elements and are found in proteins with diverse functions that also contain various other domains (Supplemental Figure 14D). Although the FANCAA element is evolutionarily ancient, there is currently no evidence for its function, and the biological significance and the reason for its evolutionary conservation remain unclear. Nevertheless, the results of our computational searches for FANCAA may support the notion that sequence shuffling or mixing is a likely mechanism by which *FAAP100* and FANCAA arose rather than de novo gene origination or gene duplication and divergence. The fact that FANCAA is only detectable in FAAP100 among the FANC proteins does not seem to support the proposal that *FAAP100,* together with *FANCA*, -*B*, -*C*, -*E*, -*F*, and -*G*, arose from a common protein at the cusp of vertebrate evolution (16, 23), but in the absence of sequence homology even between the close partners *FAAP100* and *FANCB*, parallel evolution of highly similar overall structures remains a possibility (14).

Recent structural studies of the FA core complex have made great strides in understanding the functions of FAAP100 and, in addition to an architecture consisting of WD40 repeats, coiled-coil (leucine zipper), β-sandwich, α /β, and helical bundle elements (16) have finally revealed a remarkable structural feature in FAAP100. Pairs of β-propellers have been identified at the peripheral ends of long α -helices (coiled coils) of FAAP100 and FANCB, with each pair containing a β-propeller from the N-terminal region of FAAP100 or FANCB (14). A large subset of WD40 repeat β-propellers from a functionally diverse set of proteins have been shown to bind ubiquitin and confer a variety of specific regulatory activities (41). Given that the β-propeller pairs of FAAP100 and FANCB may be capable of autoubiquitylation and that an autoubiquitylation capacity of FANCL, strongly enhanced by FAAP100, has been demonstrated both in vivo and in vitro (12, 18, 42), we hypothesized that ubiquitylation may be a common regulatory mechanism of the BLP100 subcomplex, which led us to investigate the physical interaction between FAAP100 and ubiquitin. In experiments with different antibodies and conditions, no interaction between FAAP100 and ubiquitin was detected by co-IP. However, it cannot be excluded that a potential interaction was not stabilized in our experiments, given the absence of components of the ubiquitylation cascade or factors that signal ICL damage. It may also be instructive to examine different β-propellers with respect to different ubiquitylation capacities.

The 3 proteins FANCB, FANCL, and FAAP100 form the minimal monoubiquitylation module BLP100 of the FA core complex (12). Since FANCL is known to provide the E3 ubiquitin ligase activity of the catalytic module, but is insufficient by itself in reconstituted ubiquitylation reactions (43), we investigated the contributions of FANCB and FAAP100 to the provision of ligase activity. The FANCB and FAAP100 subunits of the subcomplex with FANCL stimulate in vitro ubiquitylation activity by approximately 5- to 6-fold compared with FANCL alone (31). In reconstituted ubiquitylation reactions, the contribution of FAAP100 to the increase in activity appeared to be stronger than that of FANCB (e.g., Figure 6, A and B). It has been repeatedly shown that the deletion of any of the 3 corresponding genes in different cell types abolishes FANCD2-FANCI monoubiquitylation, while residual monoubiquitylation activity may be retained in cells deleted for other FA core complex subunits (12, 31). In survival experiments, cells deleted for *FANCB* were more sensitive than cells deleted for *FANCA*, *FANCC*, *FANCF*, or *FANCG*. Consistent with the fact that disruption of any of the BLP100 proteins completely abolishes FANCD2-FANCI monoubiquitylation in vivo and in vitro, it is conceivable that the complete absence of monoubiquitylation activity may consistently result in severe FA phenotypes or prenatal loss, as suggested by our *Faap100*-KO mouse, compared with mouse models with defects of FANCA, -C, -E, -F, or -G (36–38), and by the severe abnormalities of the fetus with the homozygous *FAAP100* mutation in the present study. This idea is reflected in the low numbers of patients with FA-B or FA-L, who represent 2% and «1% of all patients with FA, respectively (4). Since pathogenic *FANCB* mutations are hemizygous and *FAAP100* mutations are biallelic, it is conceivable that patients with FAAP100 are also less common than patients with FA-B. Patients with FA-B with disruptive *FANCB* mutations showed earlier-than-average onset of BMF and more severe congenital anomalies, including frequently the association of vertebral (V), anal atresia (A), cardiac (C), tracheo-esophageal fistula (T), esophageal/duodenal atresia (E), renal (R), and limb (L) (VACTERL) abnormalities, compared with a large series of individuals with FA (44). Similarly, single case reports of the few patients with disruptive *FANCL* mutations suggest severe phenotypes (45). Among the multiple phenotypes identified for the clinical presentation of FA, patients with *FAAAP100*, *FANCB*, or *FANCL* mutations may be best classified as having a high multiple congenital anomaly (MCA), low neoplasia phenotype due to impaired ICL repair rather than homologous recombination repair (46, 47). Considering that translocation of the entire BLP100 is FANCA dependent or that other core complex subunits may regulate the monoubiquitylation reaction in different ways, severe phenotypes and lack of monoubiquitylation are also common in patients with disruptive defects in non-BLP100 FA core complex components.

In conclusion, we have identified and characterized a homozygous pathogenic variant in *FAAP100* that leads to FA, justifying the proposal of the alias *FANCX*. Although still incomplete, we now have a remarkably detailed and nuanced picture of the FA network. However, the “never-ending story” of the ever-expanding family of FA and FA-like genes (48) is not over, as there are still gaps in our understanding of the FA network, and there are natural candidates based on their functions or interactions that could fill them in. *FAAP100* can now become an integral part of mutation screens for FA and should be considered for mutation assessment in breast and other cancers.

## Methods

### Sex as a biological variable.

Both sexes were involved in our studies. Sex was not considered as a biological variable.

Additional details on the methods used in this study can be found in Supplemental Methods.

### Cell lines.

Complete MEM, DMEM, or RPMI1640 was used for the various cultures, as recommended for the particular cell type. Primary cells and lymphoblast lines were supplemented with 15% FBS, and immortalized and established cell lines were supplemented with 10% FBS. For HEK293T cells, tetracycline-free FBS was used for lentivirus production with Tet-Off transactivation to drive high-level expression of specific packaging proteins. All cultures were maintained at 37°C in 5% CO_2_ incubators. The cell line 1176 was established from amniotic fibroblasts of the affected fetus 1176. HAP1 cell lines (HZGHC003678c001 and C631, purchased from Horizon Discovery) were cultured according to the manufacturer’s protocol. Zebrafish and mouse cell lines were established from animal tissues as described in Results. Cell immortalization, if required, was performed with large T antigen using standard procedures.

### Genome-wide SNP array.

A HumanHap300v2_A Genotyping BeatChip from Illumina was used to generate genome-wide SNP genotypes with DNA from the affected fetus, the parents, and an unaffected sibling. Comparative analysis of the genotypes was performed using AutoSNPa software developed by Ian Carr (49).

### WES.

Target enrichment and next-generation sequencing were performed as previously described (9). WES data were processed using NextGene software, version 2.4.1.2 (Softgenetics). Total reads (104,114,566 reads) were converted to FASTA format (103,369,482 reads), of which 101,858,328 reads (98.5%) were mapped to the GRCh37/hg19 reference genome assembly. The exome coverage was 196×. We found that 59.06% of the reads mapped to the region of interest (ROI) (SureSelect Human All Exon 50Mb Kit) with greater than 100× coverage. For filtering and prioritization, only variants in exons, at canonical splice sites, or in adjacent intronic sequences were considered. Variants at SNP positions (dbSNP 137; https://www.ncbi.nlm.nih.gov/snp/) and without dbSNP entry were examined separately. Given the consanguinity of the family, homozygous variants were prioritized. Genes with homozygous variants were analyzed for their potential function in DNA repair (Supplemental Table 2). Identified variants were evaluated using variant annotation, analysis, and prediction of functional effects software and were compared with databases of normal and disease-related human genetic variation (see below). Exons with low coverage were additionally analyzed by Sanger sequencing (see Supplemental Table 6 for details).

### Specialized in silico analyses.

The presence of nuclear localization signals (NLSs) in FAAP100 was examined by PSORT II. Prediction tools used to assess the pathogenicity of T542P and other sequence variants included MutationTaster, PolyPhen-2, SIFT, PROVEAN, Alamut Visual Plus, and AlphaMissense. Alignment of orthologous FAAP100 protein sequences for studies of evolutionarily conservation studies was performed using the Clustal Omega program with access to the Universal Protein Resource (UniProt). The structural effects of the missense variant T542P were assessed by Missense3D and Phyre2 with the default settings of Phyre-3-ß, using the calculated AlphaFold structural model of FAAP100, AF-Q0VG06-F1 as the template. Visualization and overlay were performed using PyMOL 3 – Schrödinger. Protein family information was obtained from the Pfam database. Protein family searches and analyses were performed using InterPro. For FANCAA studies, the NCBI Conserved Domain Architecture Retrieval Tool and the corresponding entries in the NCBI Protein database were used. Secondary protein structure predictions were generated using JPred4. FAAP100 and FANCAA alignments were generated using the ClustalX2 program.

### Plasmids.

WT *FAAP100* cDNA (NM_025161) had initially been subcloned into the pCEP4 vector (Addgene) (23). This study also used pM GAL4 DNA-BD and pVP16 AD vectors with full-length *FAAP100*, *FANCL* (NM_018062), or *FANCB* (NM_001018113) cDNA, all of which were re-used in the present study. The c.1624A>C transversion was introduced into *FAAP100* by 2-step mutagenesis (50) in a pM vector. *FAAP100^WT^* cDNA was subcloned from pCEP4 and *FAAP100* c.1624A>C cDNA from the pM vector into pLVX-EF1α -IRES-Puro vectors (Clontech). The primers used for cloning and vector analysis are listed in Supplemental Table 7.

### Gene editing.

FAAP100-deficient cell lines were generated by CRISPR/Cas9-mediated gene editing as previously described (51). Protospacer elements were designed with the CRISPR Design Tool (https://www.synthego.com/products/bioinformatics/crispr-design-tool) and subcloned into a pSpCas9(BB)-2A-Puro (PX459) vector (Addgene) using standard protocols. Single-cell colonies were isolated by serial dilution of cell suspensions in 96-well plates. *FAAP100*-edited clones were screened for *FAAP100* mutations by Sanger sequencing. Potential off-target sites were accounted for using the following prediction tools: CRISPR Design Tool (https://www.synthego.com/products/bioinformatics/crispr-design-tool), CRISPR Finder (http://www.sanger.ac.uk/htgt/wge/search_by_seq?seq_block=&bulk_species=Grch38), CHOPCHOP, version 3 (https://chopchop.cbu.uib.no/), E-CRISP (http://www.e-crisp.org/E-CRISP/), CCTOP (https://cctop.cos.uni-heidelberg.de/), BLAST (https://blast.ncbi.nlm.nih.gov/Blast.cgi), and BLAT (https://genome.ucsc.edu/cgi-bin/hgBlat?command=start). No off-target sites were predicted for the sgRNAs used, including a maximum of 3 allowed mismatches. Corresponding protospacer adjacent motifs are shown in Supplemental Table 9.

### Targeting of mouse embryonic stem cells and generation of Faap100-KO mice.

The targeting vector PRPGS00048_B_H06 from Children’s Hospital Oakland (BACPAC Resources) was linearized by digestion with PacI before electroporation into C57Bl/6 Bruce4 embryonic stem (ES) cells (52). Neomycin-resistant ES cell clones were screened by Southern hybridization to identify potentially targeted clones using a probe external to the 5′ homology arm (5P) (Supplemental Table 10). Digestion of genomic DNA with BamHI resulted in a 24.7 kb WT or a 8.2 kb targeted allele. Potentially targeted clones were further screened by Southern hybridization to confirm correct targeting as follows: 3′ integration was confirmed using a probe external to the 3′ homology arm (P3). The WT allele showed a band of 24.7 kb and the correctly targeted allele a band of 15.4 kb when genomic DNA was digested with BamHI. The possibility of additional random targeting events was excluded by using a probe to the neo cassette (NeoP). Correctly targeted alleles yielded sizes of 12.3 kb and 12.7 kb when genomic DNA was digested with HindIII and BglII, respectively. The targeting strategy is illustrated in Supplemental Figure 12A. After authentication, ES cells were injected into goGermline blastocysts (28). Chimeric mice were mated with Flp-deleter mice to remove Neo and LacZ. They were then mated with Cre-deleter mice to remove exon 3. The conditional allele and KO alleles were distinguished by RT-qPCR. Heterozygous mice were backcrossed with mice on a C57BL/6 background for 2 generations followed by heterozygous breeding to generate *Faap100^–/–^* mice (Supplemental Figure 12B).

### Mammalian 2-/3-hybrid assay.

A total of 200,000 HEK293T cells, or, in the case of 3-hybrid studies, HEK293T cells stably transfected with FLAG-tagged *FANCB* (23), were seeded in triplicate in 12-well plates. The following amounts of plasmids were cotransfected per well: 0.1 μg pM GAL4 DNA-BD (Clontech), 0.1 μg pVP16 AD cloning vector (Clontech), 0.16 μg firefly reporter plasmid pGL4.31[luc2P/CRE/Hygro] (Promega), 0.16 μg *Renilla* reporter plasmid pGL4.74[hRluc/TK] (Promega), and 0.98 μg pUC19. Plasmids pM and pVP16 contained either no cDNA or cDNA of one of the genes of interest, *FAAP100*, *FAAP100* c.1624A>C, *FANCB,* or *FANCL*. Cell lysis and luciferase activity assays were performed using the Dual-Luciferase Reporter Assay System (Promega) on a Mithras LB 940 Multimode Microplate Reader (Berthold). The fold-induction was calculated relative to the weak interaction of pM-53 and pVP16-CP. Each assay was performed as 3 independent experiments.

### Reconstitution of FANCD2 monoubiquitylation.

FAAP100^WT^/FAAP100^T542P^, FANCB, and FANCL were purified separately (Supplemental Figure 13A). A dose of 10 nM of the FANCD2-FANCI complex was incubated with 2.4 nM pBR322 DNA in a reaction buffer (10 mM HEPES pH 7.5, 1 mM MgCl_2_, 4% glycerol, 0.5 mM DTT, and 100 mM KCl) for 10 minutes at room temperature. The mixture was then incubated on ice and the following components were added: 16 nM (suboptimal) FAAP100^WT^ or FAAP100^T542P^ or otherwise indicated concentrations of FAAP100^WT^/FAAP100^T542P^, 40 nM FANCB, 10 nM UBE1, 1 nM UBE2T, 50 nM HA-tagged ubiquitin, 40 nM FANCL, and 2 mM ATP. Reaction mixtures were incubated for 4 hours at room temperature (31, 53). BLP100 complex formation was assessed by Coomassie staining and immunodetection (Supplemental Figure 13B). DNA was removed by the addition of 10 units of recombinant DNase I (Roche), followed by an incubation step of 30 minutes at 37°C. Reaction products were resolved on 8% SDS-PAGE gels. Monoubiquitylation of FANCD2 was demonstrated by the detection of the slower migrating isoform of FANCD2 (D2-Ub) by immunoblot analysis (31, 53). The ratio of FANCD2-Ub/total FANCD2 in percentage was used to quantify ubiquitylation efficiency.

### Statistics.

The numbers of experiments, clones, cells, and animals are given in the figure legends, table footnotes, and Methods. Statistical analyses for multiple comparisons were performed by 1-or 2-way repeated-measures ANOVA with post hoc analysis using Tukey’s honestly significant difference (HSD) test. Comparisons between 2 groups were conducted using the unpaired 2-sample *t* test or the Mann-Whitney *U* test in their 2-tailed versions. The specific test is identified in each figure legend. Data are presented as the mean ± SD. All statistical calculations were performed using Origin software from the OriginLab Corporation, SPSS software, Excel 2021 statistical functions, or GraphPad Prism with a significance level of 0.05. Box-and-whisker plots were generated using OriginPro 2024b software. No whiskers are shown for *n* ≤3. Figures were prepared using Adobe Illustrator or Adobe Photoshop.

### Study approval.

The research projects “Molecular Causes of Variability in Fanconi Anemia” and “Physical Mapping of Unknown Fanconi Anemia Genes” have been approved by the IRB of the Faculty of Medicine at the University of Wurzburg (23/98 and 94/04). The work of the German consortium “Translational Research for Persons with Abnormal DNA Damage Response (ADDRess),” in which RK and DS participate, has been approved by the Ethics Committee of the Hannover Medical School (no. 2850-2015). The parents of the affected fetus provided written informed consent for the cytogenetic and molecular studies. All experiments in mice were performed in accordance with protocols approved by the Ozgene Animal Ethics Committee (Bentley DC, Australia) under proposal 150602_PHEN (project 1652_Franco). Phenotypic analyses of heterozygous and homozygous *Faap100*-KO mice were performed by an Ozgene (Australia) veterinarian in accordance with Australian ethics guidelines. All zebrafish experiments were performed in accordance with the NIH guidelines for animal handling and research under a NHGRI Animal Care and Use Committee–approved (ACUC-approved) protocol (G-17-3).

### Nomenclature.

The choice of the alias *FANCX* for *FAAP100* was made by Elspeth Bruford of the HUGO Human Genome Nomenclature Committee (HGNC) and was required by the *JCI* Editorial Board.

### Data availability.

Values for all data points in graphs are reported in the Supporting Data Values file. We have also provided unedited blot and gel images in a file that contains the unedited images for all cropped blots and gels in their entirety.

## Author contributions

JK designed and performed experiments, analyzed data, prepared figures, and contributed substantially to the writing of the manuscript. YX and WW designed and performed the DT40 studies, analyzed and interpreted the data, contributed to the results, and drafted part of the manuscript. FY and YZ designed and performed the biochemical experiments, analyzed and interpreted the data, contributed to the results, and drafted part of the manuscript. RRB and SCC designed and performed the zebrafish studies, analyzed and interpreted the data, contributed to the results, and drafted part of the manuscript. SP performed, analyzed, and interpreted chromosome breakage and cell-cycle studies. RK performed and interpreted structural studies, calculated data, prepared figures and contributed to the writing of the manuscript. DS conceived the study, analyzed and interpreted data, generated results, supervised the work, and drafted the final version of the manuscript with input from the other authors.

## Supplementary Material

Supplemental data

Unedited blot and gel images

Supporting data values

## Version 1 04/15/2025

In-Press Preview

## Version 2 06/02/2025

Electronic publication

## References

[1] Kass EM (2016). When Genome Maintenance Goes Badly Awry. Mol Cell.

[2] Roger L (2021). Mechanisms and Regulation of Cellular Senescence. Int J Mol Sci.

[3] Vitale I (2017). DNA Damage in Stem Cells. Mol Cell.

[5] Dutzmann CM (2022). Cancer in children with fanconi anemia and ataxia-telangiectasia-a nationwide register-based cohort study in Germany. J Clin Oncol.

[6] Semlow DR, Walter JC (2021). Mechanisms of vertebrate DNA interstrand cross-link repair. Annu Rev Biochem.

[7] Kottemann MC, Smogorzewska A (2013). Fanconi anaemia and the repair of Watson and Crick DNA crosslinks. Nature.

[8] Oostra AB (2012). Diagnosis of fanconi anemia: chromosomal breakage analysis. Anemia.

[9] Knies K et al (2017). Biallelic mutations in the ubiquitin ligase RFWD3 cause Fanconi anemia. J Clin Invest.

[10] Catucci I (2018). Individuals with FANCM biallelic mutations do not develop Fanconi anemia, but show risk for breast cancer, chemotherapy toxicity and may display chromosome fragility. Genet Med.

[11] Bogliolo M et al (2018). Biallelic truncating FANCM mutations cause early-onset cancer but not Fanconi anemia. Genet Med.

[12] Huang Y (2014). Modularized functions of the Fanconi anemia core complex. Cell Rep.

[13] (2017). The FA core complex contains a homo-dimeric catalytic module for the symmetric mono-ubiquitination of FANCI-FANCD2. Cell Rep.

[14] Shakeel S (2019). Structure of the Fanconi anaemia monoubiquitin ligase complex. Nature.

[15] Tan W, Deans AJ (2017). A defined role for multiple Fanconi anemia gene products in DNA-damage-associated ubiquitination. Exp Hematol.

[16] Wang S (2021). Structure of the FA core ubiquitin ligase closing the ID clamp on DNA. Nat Struct Mol Biol.

[17] Meetei AR (2003). A multiprotein nuclear complex connects Fanconi anemia and Bloom syndrome. Mol Cell Biol.

[18] Meetei AR (2003). A novel ubiquitin ligase is deficient in Fanconi anemia. Nat Genet.

[19] Meetei AR (2004). X-linked inheritance of Fanconi anemia complementation group B. Nat Genet.

[20] Meetei AR (2005). A human ortholog of archaeal DNA repair protein Hef is defective in Fanconi anemia complementation group M. Nat Genet.

[21] Bythell-Douglas R, Deans AJ (2021). A structural guide to the bloom syndrome complex. Structure.

[22] Yan Z (2012). A ubiquitin-binding protein, FAAP20, links RNF8-mediated ubiquitination to the Fanconi anemia DNA repair network. Mol Cell.

[23] Ling C (2007). FAAP100 is essential for activation of the Fanconi anemia-associated DNA damage response pathway. EMBO J.

[24] Xu W (2023). FAAP100 is required for the resolution of transcription-replication conflicts in primordial germ cells. BMC Biol.

[25] Mikat B (2016). X-linked recessive VACTERL-H due to a mutation in FANCB in a preterm boy. Clin Dysmorphol.

[26] Jeon S (2020). Korean Genome Project: 1094 Korean personal genomes with clinical information. Sci Adv.

[27] Ramanagoudr-Bhojappa R (2018). Multiplexed CRISPR/Cas9-mediated knockout of 19 Fanconi anemia pathway genes in zebrafish revealed their roles in growth, sexual development and fertility. PLoS Genet.

[28] Koentgen F (2016). Exclusive transmission of the embryonic stem cell-derived genome through the mouse germline. Genesis.

[29] Kalter H (1968). Sporadic congenital malformations of newborn inbred mice. Teratology.

[30] Yuan F (2009). FANCI protein binds to DNA and interacts with FANCD2 to recognize branched structures. J Biol Chem.

[31] Rajendra E (2014). The genetic and biochemical basis of FANCD2 monoubiquitination. Mol Cell.

[32] Howe K (2013). The zebrafish reference genome sequence and its relationship to the human genome. Nature.

[33] Titus TA (2009). The Fanconi anemia/BRCA gene network in zebrafish: embryonic expression and comparative genomics. Mutat Res.

[34] Rodríguez-Marí A (2011). The role of Fanconi anemia/BRCA genes in zebrafish sex determination. Methods Cell Biol.

[35] Yang Y (2019). RNA 5-methylcytosine facilitates the maternal-to-zygotic transition by preventing maternal mRNA decay. Mol Cell.

[36] Bakker ST (2013). Learning from a paradox: recent insights into Fanconi anaemia through studying mouse models. Dis Model Mech.

[37] Dong H (2015). Update of the human and mouse Fanconi anemia genes. Hum Genomics.

[38] Guitton-Sert L (2021). Animal models of Fanconi anemia: a developmental and therapeutic perspective on a multifaceted disease. Semin Cell Dev Biol.

[39] Tomaszowski KH (2023). Hypomorphic Brca2 and Rad51c double mutant mice display Fanconi anemia, cancer and polygenic replication stress. Nat Commun.

[40] Fakhar AZ (2023). The lost and found: unraveling the functions of orphan genes. J Dev Biol.

[41] Pashkova N (2010). WD40 repeat propellers define a ubiquitin-binding domain that regulates turnover of F box proteins. Mol Cell.

[42] Longerich S (2009). FANCI binds branched DNA and is monoubiquitinated by UBE2T-FANCL. J Biol Chem.

[43] Alpi AF (2008). Mechanistic insight into site-restricted monoubiquitination of FANCD2 by Ube2t, FANCL, and FANCI. Mol Cell.

[44] Jung M (2020). Association of clinical severity with FANCB variant type in Fanconi anemia. Blood.

[45] Wu W (2017). Novel homozygous FANCL mutation and somatic heterozygous SETBP1 mutation in a Chinese girl with Fanconi Anemia. Eur J Med Genet.

[46] Neveling K (2009). Genotype-phenotype correlations in Fanconi anemia. Mutat Res.

[47] Wang AT (2015). A dominant mutation in human RAD51 reveals its function in DNA interstrand crosslink repair independent of homologous recombination. Mol Cell.

[48] Gueiderikh A (2017). A never-ending story: the steadily growing family of the FA and FA-like genes. Genet Mol Biol.

[49] Carr IM (2006). Interactive visual analysis of SNP data for rapid autozygosity mapping in consanguineous families. Hum Mutat.

[50] Wang W, Malcolm BA (2002). Two-stage polymerase chain reaction protocol allowing introduction of multiple mutations, deletions, and insertions, using QuikChange site-directed mutagenesis. Methods Mol Biol.

[51] Ran FA (2013). Genome engineering using the CRISPR-Cas9 system. Nat Protoc.

[52] Kontgen F (1993). Targeted disruption of the MHC class II Aa gene in C57BL/6 mice. Int Immunol.

[53] Longerich S (2014). Regulation of FANCD2 and FANCI monoubiquitination by their interaction and by DNA. Nucleic Acids Res.

